# Synthesis of Chitosan based nanoemulsions and their characterization and antifungal activity toward fungi causing mucormycosis

**DOI:** 10.1038/s41598-025-06577-7

**Published:** 2025-06-27

**Authors:** Mohamed S. Hasanin, Fathallah A. Ayoob, Amr H. Hashem, Mahmoud Emam

**Affiliations:** 1 Cellulose & Paper Department, National Research Centre, El-Buhouth St., Dokki, 12622 Egypt; 2 Chemical industries Research Institute, National Research Centre, Dokki, Giza, 12622 Egypt; 3 Botany and Microbiology Department, Faculty of Science, Al-Azhar University, Cairo, 11884 Egypt; 4 Phytochemistry and Plant Systematics Department, National Research Centre, Dokki, Giza, 12622 Egypt

**Keywords:** Antifungal activity, Fungal infection, Mucormycosis, Nanoemulsion, Nanochitosan, Phytochemical analysis, Drug discovery, Microbiology

## Abstract

Aromatic plants produce essential oils (EOs) with diverse phytochemicals and biological applications. This study investigated three eco-friendly nanoemulsions of Lemon peel (LPO), Turmeric (TO), and Black seed (BSO) oils loaded into nanochitosan (NCh) for their antifungal activity against resistant fungal strains. Phytochemical analysis identified oxygenated/non-oxygenated hydrocarbons and saturated/unsaturated fatty acids in the EOs. Physicochemical characterization using FTIR, DLS, and HR-TEM showed stable nanoemulsions and nanochitosan with homogeneous particle size distributions in the nanoscale range. Notably, the essential oil nanoemulsions exhibited potent antifungal activity against *Mucor racemosus*,* Rhizopus microsporus*, and *Lichtheimia corymbifera*, resistant to commercial antifungal drugs. The nanoemulsions loaded with 1–3% chitosan showed inhibition zones ranging from 17 to 23 mm, outperforming the synthetic antifungal treatments. These findings highlight the potential of plant-derived essential oil nanoemulsions loaded into biocompatible nanochitosan as a promising, sustainable alternative to combat the growing threat of invasive fungal infections and drug resistance. Incorporating natural, eco-friendly materials enhances the stability, bioavailability, and targeted delivery of the active phytochemicals, contributing to the antifungal solution’s overall efficacy and safety profile.

## Introduction

Invasive fungal infections have been a growing concern in recent years, with certain types posing significant health risks. One such infection is mucormycosis, caused by a group of fungi called mucormycetes. Mucormycosis is an opportunistic infection that primarily affects individuals with weakened immune systems, such as those with uncontrolled diabetes, cancer, organ transplants, or those taking immunosuppressive medications^1^. The infection can manifest in various forms, including rhino-cerebral (sinus, nose, and brain), pulmonary (lung), cutaneous (skin), and disseminated (spread throughout the body)^2^. Mucormycosis is a rapidly progressive and potentially life-threatening condition requiring prompt diagnosis and aggressive treatment with antifungal medications and, in some cases, surgical intervention^3^. In addition to mucormycosis, other invasive fungal infections, such as aspergillosis, candidiasis, and cryptococcosis, have also been observed with increasing frequency in recent years. These infections can be challenging to diagnose and treat, as they often present with nonspecific symptoms and can be difficult to distinguish from bacterial or viral infections^4,5^. Ongoing research and development of new antifungal therapies are crucial to addressing the growing burden of invasive fungal infections and improving patient outcomes^6–9^.

Plant-derived essential oils have been utilized for numerous purposes and have a significant history as natural food preservatives, therapeutic agents, medicinal substances, and healing modalities^10^. According to many sources, plants’ essential oils (EOs) have antioxidant, antiradical, and antimicrobial properties^11^. They are now commonly used as functional ingredients in the food and pharmaceutical industries^12,13^. EOs are limited due to their inability to dissolve in water. Thus, oil-in-water or nano-emulsions improve their solubility^14,15^. EOs are versatile and contain biological substances that have the potential for use in nutrition and pharmaceutical companies as supplements or antibacterial agents. They can also improve the shelf life of grains and seeds^16^. It has been documented that EOs have important antiseptic, antimicrobial, antiviral, antifungal, and insecticide activity^17–21^.

The antifungal properties of plant-derived essential oils are promising and can be improved by utilizing nanotechnology^22^. Nanoemulsions are a type of nanoformulation that can improve the pharmacological effects of EOs^23^. This also enhances their antimicrobial effectiveness by increasing the surface area of contact with microorganisms^24^. Nanoemulsions of essential oils were tested for their antifungal properties against different fungal strains, such as *Penicillium digitatum*^23,25^. Among these, lemon oil nanoemulsion (LO-NE) has potential as an antifungal agent. It is physically stable with a small particle size. LO-NE has better antioxidant activity than pure lemon oil. In addition, the stability analysis showed that LO-NE outperformed pure lemon oil. LO-NE may be helpful for transdermal disorders^26–28^. Also, curcumin essential oil nanoemulsions have been studied for their antifungal activity. Essential oil nanoemulsions, including *P. digitatum*, can inhibit fungal growth with low MIC. Therefore, curcumin essential oil nanoemulsions have antifungal potential for controlling fungal infections^29^. Curcumin, a bioactive compound in turmeric, has various health benefits, including antioxidant, anticancer, and anti-inflammatory effects. These properties promote the use of curcumin in health-improving food and supplement products^30^. Curcumin undergoes degradation when exposed to light and is unstable at human physiological pH. Moreover, it lacks solubility in water^31^.

Furthermore, black seed oil is from the seeds of the *Nigella sativa* plant and has been used for medicinal purposes for many years. As several references mentioned, it has potential therapeutic properties, highlighting the potential benefits of black seed oil^32^. Black seed essential oil nanoemulsions have potential as an antifungal agent^22^. To improve the chemical stability, bioavailability, and solubility of EOs in a suitable delivery system, the encapsulation of EOs is the desired approach, such as an emulsion^33^. Nanoemulsions with a negative charge containing black seed essential oil showed prolonged antibacterial effectiveness^34^.

Formulation of the emulsion also plays a role in their stability and efficiency^35,36^. Polysaccharides are attractive materials for applications with unique characteristics and features such as multifunctionality, biodegradability, cytocompatibility, and nontoxicity. Chitosan is a polysaccharide containing amino groups that give it reactivity and sometimes antimicrobial activity^34^. NCh has all the characteristics of chitosan with small particle sizes and could stabilize the nanoemulsion formulas.

This study was constructed to illustrate a comparative survey of eco-friendly nanoemulsions of Lemon peel, Turmeric, and Black seed oils that are categorised as generally recognised as safe (GRAS) oils loaded into nanochitosan. Phytochemical, physicochemical, and morphological analyses were conducted to evaluate the formulations. Antifungal activity was also assessed for its efficiency against fungi causing mucormycosis, such as *M. racemosus*, *R. microsporus*, and *L. corymbifera.*

## Materials and methods

### Materials

Chitosan with a molecular weight of 20 kDa and a deacetylation degree higher than 85% was purchased from Sigma Aldrich (Austria). Loba Chem, India, purchased sodium tripolyphosphate (TPP) and Tween 80. All chemicals and solvents were HPLC or GC grade.

### Plant material

Three different oils, *Citrus limon* (peel), *Curcuma longa* L. (rhizome), and *Nigella sativa* L. (seed) (Table 1), were obtained from the National Research Centre’s pressing and extraction unit.

**Table 1:** The plant used in the study.

Common name	Scientific name	Abbreviations	Extraction method
Lemon peel oil	*Citrus limon* oil	LPO	Hydrodistillation
Turmeric oil	*Curcuma longa* L. oil	TO	Hydrodistillation
Black seed oil	*Nigella sativa* L. oil	BSO	Hydraulic Cold Press

### Phytochemical analysis of the selected oils

The sample was saponified with ethanolic potassium hydroxide, and an unsaponifiable (UNSAP) fraction was extracted in petroleum ether. While the saponified fraction extracted in petroleum ether was mixed with 50 µL of bis(trimethylsilyl)trifluoroacetamide (BSTFA) + trimethylchlorosilane (TMCS) 99:1 sialylation reagent, and 50 µL pyridine were used to derivatize sample functional groups to trimethylsilyl groups (abbreviated TMS) before GC analysis.

#### Gas chromatography–mass spectrometry analysis (GC–MS)

The GC-MS system (Agilent Technologies) was equipped with a gas chromatography (7890B) and mass spectrometer detector (5977 A) at Central Laboratories Network, National Research Centre, Cairo, Egypt. The GC had an HP-5MS column (30 m x 0.25 mm internal diameter and 0.25 μm film thickness). Analyses were carried out using Hydrogen as the carrier gas at a flow rate of 2.0 ml/min at a splitless injection volume of 2 µl and the following temperature program: 50 °C for 5 min; rising at 5 °C/min to 100 °C and held for 0 min and rising at 10 °C/min to 320 °C and held for 10 min. The injector and detector were held at 280 °C and 320 °C, respectively. Mass spectra were obtained by electron ionization (EI) at 70 eV, using a spectral range of m/z 25–700 and a solvent delay of 6 min. The mass temperature was 230 °C and Quad 150 °C. Different constituents were identified by comparing the spectrum fragmentation pattern with those stored in the Wiley and NIST Mass Spectral Library data.

#### Gas chromatography for fatty acids methyl ester (FAME)

The GC model7890B from Agilent Technologies was equipped with a flame ionization detector at Central Laboratories Network, National Research Centre, and Cairo, Egypt. Separation was achieved using a Zebron ZB-FAME column (60 m x 0.25 mm internal diameter x 0.25 μm film thickness). Analyses were carried out using hydrogen as the carrier gas at a flow rate of 1.8 ml/min at a split-1:50 mode, injection volume of 1 µl, and the following temperature program: 100 °C for 3 min, rising at 2.5 °C/min to 240 °C and held for 10 min. The injector and detector (FID) were held at 250 °C and 285 °C, respectively.

### Nano-preparation

#### Nanochitosan preparation

A chitosan solution is prepared by dissolving 2 g in a 1% (*w/w*) acetic acid solution. The solution is stirred at 1500 rpm for 3 h. Afterward, TPP is added to the solution at 100 mg/100 mL and stirred overnight at 40 °C ^37^. The above-collected solution is ultrasonicated for 10 min. The produced NCh is preserved in the refrigerator for further work.

#### Preparation of essential oil nanoemulsion

The nanoemulsion of three essential oils derived from Lemon peel, Turmeric, and Black seed or black cumin was formulated slightly in a method given by Sugumar et al.^38^. The oil-in-water nanoemulsion was formulated using an essential oil, a non-ionic surfactant (Tween 80), and water. The concentration of each essential oil was fixed for all the formulations to the surfactant with a ratio of 1:0.2. Initially, the coarse emulsion was prepared by adding essential oil at a slow rate of one ml per minute to the organic phase containing water and surfactant (0.2% *w/v*) using a magnetic stirrer at 1500 rpm and kept stirring overnight at room temperature. Each oil emulsion sample was prepared using a typical methodology without adding NCh as a blank, called LPO 0, TO 0, and BSO 0.

#### Preparation of Nanochitosan loaded nanoemulsions

**Scheme 1:**
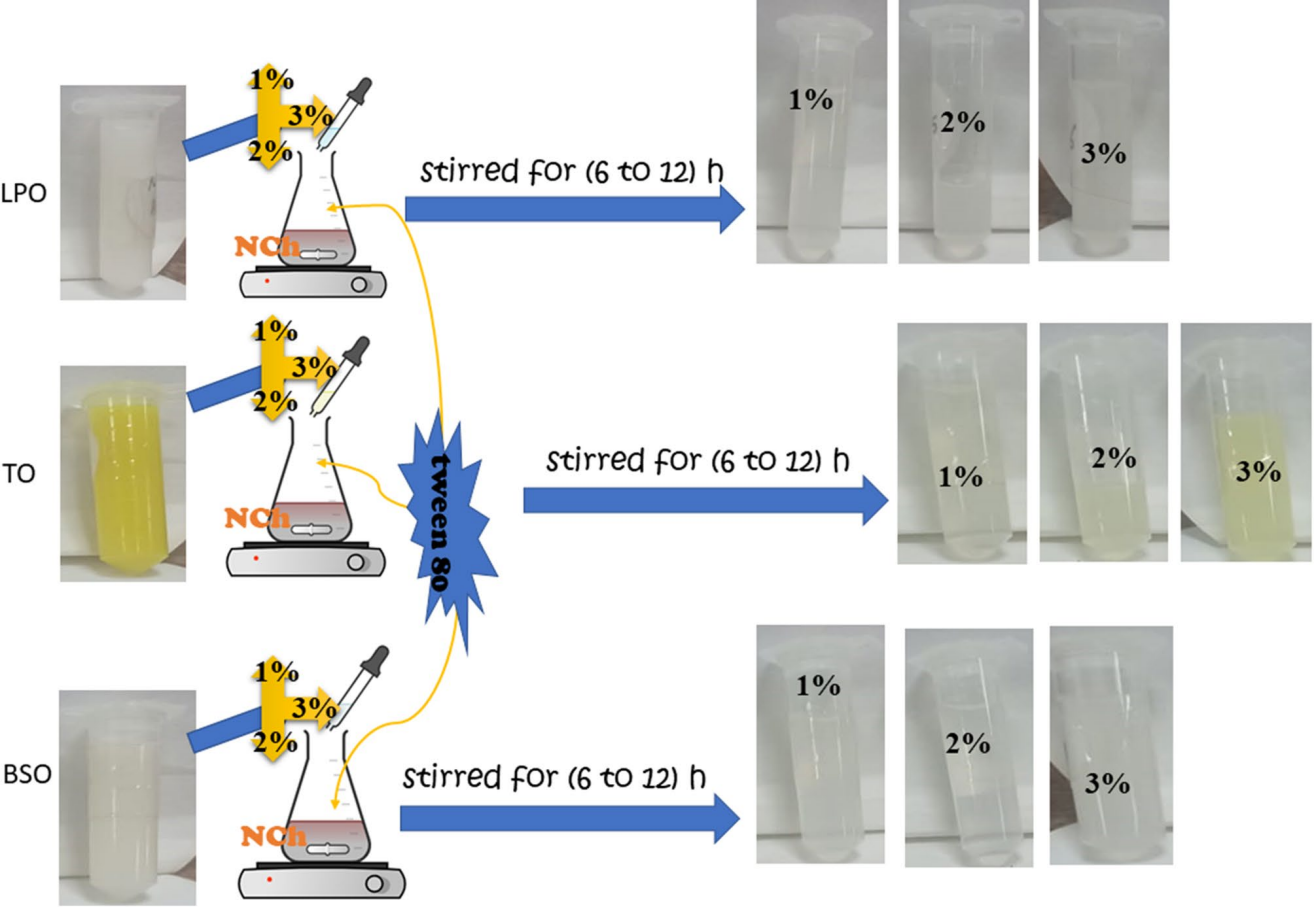
Preparation of nanoemulsion essential oil-loaded Nch.

NCh was used as a carrier of the above-prepared emulsions, as illustrated in Scheme 1. After that, different stable ratios of oil in water of LPO, TO, and BSO were prepared as shown in Table 2 and mixed with NCh solution in three different ratios for each oil individually. The prepared LPO, TO, and BSO emulsions were added to the NCh solution according to the method described by Keawchaoon and Yoksan^39^ with some modifications. In a brief description, the essential oils LPO, TO, and BSO, as the oily phase at (1%, 2%, and 3% (w/v)) concentrations, were mixed with 0.2% (w/v) of Tween 80 as a surfactant until a homogeneous mixture was obtained. Then, the mixture was added to the prepared NCh and stirred for another 6 to 12 h. These solutions were homogenized at 1500 rpm for 6 h to ensure emulsion formation and to stabilize nanoparticles.

**Table 2:** Loaded nanoemulsion composition.

Oil	Oil concentration (w/v) (%)	Tween 80 (w/v) (%)	Nanochitosan (v/v) (%)	Distilled water (v/v) (%)
LPO	1	0.2	10	88.8
	2			87.8
	3			86.8
TO	1	0.2	10	88.8
	2			87.8
	3			86.8
BSO	1	0.2	10	88.8
	2			87.8
	3			86.8

### Characterizations

FTIR (Thermo Fisher Scientific, Nicolet iS5 FT-IR spectrometer, Waltham, MA, USA). Each spectrum was the averaged value of 20 scans in transmittance mode from 4000 to 600 cm^−1^ at a resolution of 4 cm^−1^. Results were reported in normalized transmittance mode. Scanning electron microscopy (SEM) (JSM 6360LV, EOL/Noran) was used to examine the surface morphology of the prepared samples attached to the EDX analyzer. A high-resolution transmission electron microscope HR-TEM, Model JEM2010, Japan) was used to estimate the particle size and morphology of the samples. The TEM image process and particle size counting were done using ImageJ, free software. Dynamic light scattering (DLS) instrument (Santa Barbara, CA, USA) that was utilized to ascertain the average particle size distribution in nm and average zeta potential in mV, in conditions 23 °C, with the incident light being the 632.8 nm line of a HeNe laser at an angle of 13.9°). The DLS measurements were carried out in triplicate, and the recorded values of the main with the standard deviation.

### In vitro activity

#### Cytotoxicity

The MTT experimental procedure^40^ assessed the cytotoxicity of LPO 3%, TO 3%, BSO 3%, and Nch. The cell line Wi38 was obtained from the American Type Culture Collection (ATCC). The cell count and the proportion of viable cells were calculated using the following formula:

$$\:\text{V}\text{i}\text{a}\text{b}\text{i}\text{l}\text{i}\text{t}\text{y}\:\text{\%}=\:\frac{\text{T}\text{e}\text{s}\text{t}\:\text{O}\text{D}}{\text{C}\text{o}\text{n}\text{t}\text{r}\text{o}\text{l}\:\text{O}\text{D}}\:\times\:\:100$$

#### Antifungal susceptibility of some mucorales toward commercial antifungal discs

Five commercial antifungal discs (Clotrimazole 50 µg/disc, Nystatin 100 unit /disc, Econazole 10 µg/disc, Voriconazole 1 unit/disc, Ketoconazole 10 µg/disc) were used against *Mucor racemosus* (accession no MG547571.1), *Rhizopus microsporus* (accession no MK623262.1), and *Lichtheimia corymbifera* (accession no MK300698.1). These fungal strains were cultured on SDA, and all discs were placed on the surface of the plate, and the plates were incubated at 30 °C for 2 days. The diameter of the inhibition zones around the antifungal discs was measured and recorded. The inhibition zone diameters were interpreted using the established or clinical breakpoints for each antifungal agent and Mucorales species, as shown in Table 3.

**Table 3:** Interpretive guidelines on antifungal susceptibility testing.

Antifungal drug	Potency	Zone diameter/mm		
		S	S-DD	*R*
Clotrimazole	50 µg/disc	≥ 20	15–19	≤ 14
Nystatin	100 units/disc	≥ 20	15–19	≤ 14
Econazole	10 µg/disc	≥ 20	15–19	≤ 14
Ketoconazole	10 µg/disc	≥ 20	15–19	≤ 14
Voriconazole	1 unit/disc	≥ 17	14–16	≤ 13

*S* susceptible, *S-DD* susceptible dose-dependent, *R* resistant.

#### Antifungal activity of all prepared nanoemulsions using agar well diffusion method

The antifungal activity of LPO (1, 2 & 3%), TO (1, 2 & 3%), BSO (1, 2 & 3%), and Nch was evaluated using agar well diffusion against M. racemosus, R. microsporus, and *L. corymbifera*. All tested fungal strains were grown on PDA plates and incubated for 2–3 days at 30 °C^41,42^. The fungal suspension was prepared in sterilized phosphate buffer solution (PBS), pH 7.0, and then the inoculum was adjusted to 10^7^ spores/mL after counting in a cell counter chamber. One milliliter was uniformly distributed on agar MEA plates. Using a sterile cork-borer, wells (8 mm) were cut; 100 µL of LPO (1, 2 & 3%), TO (1, 2 & 3%), BSO (1, 2 & 3%), Nch were transferred to each well individually and left for 2 h at 4 °C, then all plates were incubated for 3 days at 30 °C. After incubation, the inhibition zones were determined and recorded.

### Statistical analysis

The data in each table is shown as mean ± SE and calculated from three replicates. (Dr. Amr kindly, insert further analysis that was used, if possible).

## Results and discussion

### Phytochemical analysis

The hydrocarbons and fatty acid content were identified by GS-MS and -FID, respectively. The data illustrated in Tables 4 and 5 revealed that lemon peel oil (LPO) contains eight components classified as alcoholic monoterpenoids and saturated fatty acids. Dihydromyrcenol structure was the most predominant at 44.45%, while (+)-Carvomenthol was the lowest at 4.37%.

**Table 4:** Chemical compositions of UNSAP of LPO identified by GC-MS analysis.

No.	Rt (min)	RRT	Compound identified	Molecular formula	Area sum %	Class
1	8.309	1	Dihydromyrcenol	C_10_H_20_O	44.45	OH
2	9.166	1.1	Linalool	C_10_H_18_O	30.22	OH
3	12.187	1.5	(+)-Carvomenthol	C_10_H_18_O	4.37	OH
4	16.634	2	Geraniol	C_10_H_18_O	20.96	OH
Rel % Total					100%	

Relative percent % of total identified compounds 100%, RRT: retention time relative to that of Dihydromyrcenol (Rt = 29.43 min), OH; oxygenated hydrocarbon.

Moreover, the data illustrated in Table 5 showed that the saturated fatty acid of Stearic acid is the most representative one, with 48.46% according to their peak areas.

**Table 5:** Chemical compositions of fatty acids of LPO identified by GC-FID analysis.

Peak	RT	RRT	Name	Area sum %	Class
1	29.5	0.8	Palmitic acid	47.48	Saturated fatty acid
2	35.1	1	Stearic acid	48.46	Saturated fatty acid
3	40.5	1.26	Arachidic acid	2.54	Saturated fatty acid
4	45.41	1.29	Behenic acid	1.52	Saturated fatty acid
Rel % Total				100%	

*Rel%* % of total identified compounds 100%, *RRt* retention time relative to Stearic acid (Rt = 35.1 min).

The LPO oil contained eight components that fall into two chemical categories. The first one is alcoholic monoterpenoids: these are C10 compounds derived from two isoprene units and include an alcohol functional group (–OH). They are common in essential oils and contribute to fragrance and bioactivity. For instance, Dihydromyrcenol (44.45%) is a monoterpene alcohol with a pleasant scent widely used in perfumery. (+)-Carvomenthol (4.37%): another monoterpenoid alcohol with potential antimicrobial properties. The second one is saturated fatty acids: long-chain carboxylic acids without double bonds, typically solid at room temperature. The most abundant among these was Stearic Acid (C18:0), with a peak area representing 48.46% of total fatty acids, indicating it is a major lipid constituent in LPO. The dominance of dihydromyrcenol suggests LPO has strong fragrance and potential antimicrobial properties, which may be valuable in cosmetic or pharmaceutical formulations^43,44^. The high proportion of stearic acid points to a significant lipidic or waxy component in the oil, potentially influencing texture and stability in product applications (e.g., soaps, emulsions)^45^. The presence of volatile monoterpenoids and non-volatile fatty acids in the oil indicates a chemically diverse profile, likely contributing to aroma and biofunctional properties.

In addition, the data illustrated in Tables 6 and 7 revealed that turmeric oil (TO) contains 37 components, which are classified as hydrocarbons (oxygenated or non-oxygenated) and saturated fatty acids (saturated and unsaturated).

**Table 6:** Chemical compositions of UNSAP of to identified by GC-MS analysis.

No.	RT	RRT	Compound name	Molecular formula	Area sum %	Class
1	16.55	0.48	Tetracyclo[3.3.1.0.1(3,9)]decan-10-one	C_10_H_12_O	6.48	OH
2	16.75	0.49	α,α-Dimethylbenzenepropanoic acid ethenyl ester	C_13_H_16_O_2_	12.85	OH
3	17.16	0.50	megastigma-3,7(E),9-triene	C_13_H_20_	6.69	HC
4	18.42	0.54	Dihydrocurcumene	C_15_H_22_	2.32	HC
5	18.62	0.54	β-curcumene	C_15_H_24_	1.44	HC
6	19.00	0.55	Aromandendrene	C_15_H_24_	2.10	HC
7	20.89	0.61	β-Tumerone	C_15_H_22_O	9.11	OH
8	21.27	0.62	α-Tumerone	C_15_H_22_O	3.28	OH
9	26.01	0.76	Phytol	C_20_H_40_O	2.38	OH
10	26.58	0.77	Undec-10-yonic acid, dodecyl ester	C_23_H_42_O_2_	0.58	OH
11	26.69	0.78	1-Heptatriacotanol	C_37_H_76_O	1.85	OH
12	29.42	0.86	1-Monopalmitin	C_19_H_38_O_4_	1.03	OH
13	30.81	0.90	Monostearin	C_21_H_42_O_4_	1.50	OH
14	30.98	0.90	2-Dodecen-1-yl(-)succinic anhydride	C_16_H_26_O_3_	0.61	OH
15	32.19	0.94	Ɣ-Tocopherol	C_28_H_48_O_2_	1.65	OH
16	33.08	0.96	α.-Tocopherol	C_28_H_48_O_2_	1.12	OH
17	33.76	0.98	Campesterol	C_28_H_48_O	6.21	OH
18	33.94	0.99	Stigmasterol	C_29_H_48_O	1.97	OH
19	34.33	1.00	(3β,24 S)-stigmast-5-en-3-ol	C_29_H_50_O	33.18	OH
20	34.40	1.00	Gorgosterol	C_30_H_50_O	2.70	OH
21	34.58	1.01	Cholesta-8,24-dien-3-ol, 4-methyl-, (3β.,4α)-	C_28_H_46_O	0.96	OH
			Rel % total		100%	

*OH* oxygenated hydrocarbon, *HC* hydrocarbon, Relative percent % of total identified compounds 95.84%, *rRT* retention time relative to Rt = 34.33 min.

**Table 7:** Chemical compositions of fatty acids of to identified by GC-FID analysis.

Peak	RT	RRT	Name	Area sum %	Class
1	20.67	0.55	Tridecanoic acid	0.05	Saturated fatty acid
2	29.62	0.78	Palmitic acid	8.53	Saturated fatty acid
3	30.76	0.81	Palmitoleic acid	0.09	Monounsaturated fatty acid
4	32.42	0.86	Margaric acid	0.03	Saturated fatty acid
5	33.42	0.88	cis-10-Heptadecenoic acid	0.02	Monounsaturated fatty acid
6	35.29	0.93	Stearic acid	5.21	Saturated fatty acid
7	36.15	0.95	Oleic acid	36	Monounsaturated fatty acid
8	37.86	1	Linoleic acid	48.22	Polyunsaturated fatty acid
9	39.82	1.05	Linolenic acid	0.62	Polyunsaturated fatty acid
10	40.41	1.07	Arachidic acid	0.5	Saturated fatty acid
11	41.11	1.09	*cis*-11-Eicosenoic acid	0.17	Monounsaturated fatty acid
12	42.67	1.12	*cis*-11,14-Eicosadienoic acid	0.01	Polyunsaturated fatty acid
13	45.34	1.19	Behenic acid	0.22	Saturated fatty acid
14	45.94	1.21	Erucic acid	0.2	Monounsaturated fatty acid
15	47.70	1.26	Tricosanoic acid	0.02	Saturated fatty acid
16	49.98	1.32	Lignoceric acid	0.1	Saturated fatty acid
Rel % Total				100	

*Rel%* % of total identified compounds 100%, *rRt* retention time relative to Linoleic acid (Rt = 37.865 min).

Generally, the chemical diversity of To indicates that the oil is not just a simple essential oil but a multifunctional complex with potential uses in health, cosmetics, and pharmaceuticals. The hydrocarbons are diverse and include: non-oxygenated hydrocarbons such as megastigma-3,7(E),9-triene, dihydrocurcumene, and β-curcumene. These are typically volatile terpenes or sesquiterpenes, contributing to aroma and potential biological activity. In addition, oxygenated hydrocarbons such as β-tumerone, α-tumerone, and aromandendrene. These compounds often exhibit enhanced biological properties like antimicrobial or anti-inflammatory effects due to their polar functional groups (e.g., ketones, alcohols)^46^.

In addition, fatty acid derivatives and ester compounds such as 1-Monopalmitin and Monostearin are monoacylglycerols that suggest partial hydrolysis or esterification of triglycerides. Undec-10-ynoic acid, dodecyl ester, and α,α-Dimethylbenzenepropanoic acid ethenyl ester are esters in fragrance or emulsifying functions. 2-Dodecen-1-yl(-)succinic anhydride is a reactive anhydride, possibly a minor component or additive with emulsifying or surfactant properties^47^.

Furthermore, phytol is a diterpene alcohol from chlorophyll degradation, often linked to antioxidant and anti-inflammatory activities^48^. 1-Heptatriacotanol is a long-chain alcohol that may function in skin-conditioning or barrier-forming roles^49^. Tocopherols (γ- and α-) are forms of vitamin E with strong antioxidant activity^50^. While, campesterol, stigmasterol, gorgosterol, and cholesta-8,24-dien-3-ol, are sterols. These plant-derived sterols contribute to membrane stability and may lower cholesterol levels when consumed^51^.

The fatty acids detected in Table 7 included tridecanoic (C13), palmitic (C16:0), margaric (C17:0), stearic (C18:0), arachidic (C20:0), behenic (C22:0), tricosanoic (C23:0), lignoceric (C24:0), these saturated fatty acids contribute to stability and long shelf life, as they are less prone to oxidation^52^. While palmitoleic (C16:1), cis-10-Heptadecenoic (C17:1), oleic (C18:1), linoleic (C18:2), linolenic (C18:3), cis-11-eicosenoic (C20:1), cis-11,14-eicosadienoic (C20:2), and erucic (C22:1) were considered unsaturated fatty acids. These unsaturated fatty acids provide functional health benefits, such as cardiovascular protection^53^, anti-inflammatory activity^54^, and skin nourishment^55^.

The data illustrated in Tables 8 and 9 revealed that the *Nigella sativa* seed oil (BSO) contains 35 components classified as oxygenated hydrocarbons, non-oxygenated hydrocarbons, unsaturated fatty acids, and saturated fatty acids. In which (3*β*, 24*S*)-stigmast-5-en-3-ol structure illustrated the most predominant existence, with 27.48% as an oxygenated hydrocarbon structure, while Linoleic acid represented the highest fatty acid “unsaturated” percent, with 56.98%.

**Table 8:** Chemical compositions of UNSAP of BSO identified by GC-MS analysis.

Peak	RT	rrt	Compound name	Formula	Area sum %	Class
1	8.64	0.25	2(2-Hydroxy)ethoxyethanol	C_4_H_10_O_3_	2.36	OH
2	9.11	0.27	4-Ethyl-1-octyn-3-ol	C_10_H_18_O	2.00	OH
3	9.46	0.28	1,6-Dihydrocarveol	C_10_H_18_O	3.74	OH
4	16.94	0.49	Longifolene	C_15_H_24_	2.69	HC
5	23.72	0.69	Methyl 10,12-pentacosadiynoate	C_26_H_44_O_2_	1.88	OH
6	27.27	0.79	Undecane, 1,2-dibromo-2-methyl-	C_12_H_24_Br_2_	1.31	HC
7	28.32	0.83	2-*tert*-butyl-6-methylphenol	C_11_H_16_O	3.67	OH
8	28.84	0.84	2,2’-Methylenebis(6-tert-butyl-p-cresol)	C_23_H_32_O_2_	10.62	OH
9	29.42	0.86	1-Monopalmitin	C_19_H_38_O_4_	2.93	OH
10	30.80	0.89	Monostearin	C_21_H_42_O_4_	2.17	OH
11	30.98	0.90	trans-Farnesol	C_15_H_26_O	2.05	OH
12	33.73	0.98	Campesterol	C_28_H_48_O	4.83	OH
13	33.93	0.99	Stigmasterol	C_29_H_48_O	5.47	OH
14	34.3	1	(3β,24 S)-stigmast-5-en-3-ol	C_29_H_50_O	30.59	OH
15	34.38	1.00	4α-methyl-Cholesta-8,24-dien-3β-3-ol	C_28_H_46_O	7.78	OH
16	34.63	1.00	9,19-Cyclolanost-24-en-3β-3-ol	C_30_H_50_O	11.55	OH
17	34.96	1.02	9,19-Cyclo-9β-lanostan-3β-ol	C_31_H_52_O	4.34	OH
Rel % Total					100	

Relative percent % of total identified compounds 100%, *rRT* retention time relative to Rt = 34.3 min.

**Table 9:** Chemical compositions of fatty acids of BSO identified by GC-FID analysis.

Peak	RT	rrt	Name	Area sum %	Class
1	23.68	0.62	Myristic acid	0.09	Saturated fatty acid
2	26.67	0.70	Pentadecanoic acid	0.02	Saturated fatty acid
3	29.62	0.78	Palmitic acid	11.79	Saturated fatty acid
4	30.76	0.81	Palmitoleic acid	0.17	Unsaturated fatty acid
5	32.41	0.85	Margaric acid	0.06	Saturated fatty acid
6	33.40	0.88	cis-10-Heptadecenoic acid	0.03	Unsaturated fatty acid
7	35.24	0.93	Stearic acid	3.07	Saturated fatty acid
8	36.09	0.95	Oleic acid	24.25	Unsaturated fatty acid
9	37.86	1	Linoleic acid	56.98	Unsaturated fatty acid
10	39.81	1.05	Linolenic acid	0.31	Unsaturated fatty acid
11	40.40	1.06	Arachidic acid	0.2	Saturated fatty acid
12	41.10	1.08	*cis*-11-Eicosenoic acid	0.3	Unsaturated fatty acid
13	42.67	1.12	*cis*-11,14-Eicosadienoic acid	2.51	Unsaturated fatty acid
14	44.26	1.16	Arachidonic acid	0.03	Unsaturated fatty acid
15	45.33	1.19	Behenic acid	0.03	Saturated fatty acid
16	45.93	1.21	Erucic acid	0.09	Unsaturated fatty acid
17	47.34	1.25	*cis*-13,16-Docosadienoic acid	0.03	Unsaturated fatty acid
18	49.97	1.32	Lignoceric acid	0.02	Saturated fatty acid
			Rel % Total	100	

*Rel%* % of total identified compounds ≈ 100%, *rRt* retention time relative to Linoleic acid (Rt = 37.861 min).

The oxygenated and non-oxygenated hydrocarbon compounds mentioned in Tables 8 and 9 were characterized with compounds containing oxygen-bearing functional groups (e.g., alcohols, phenols). These compounds enhance reactivity and biological properties such as antioxidant, anti-inflammatory, or antimicrobial effects, such as (3β,24 S)-Stigmast-5-en-3-ol (27.48%), the most dominant sterol, which structurally resembles cholesterol and is known for its cholesterol-lowering effect^56–58^. Trans-Farnesol and 1,6-Dihydrocarveol are oxygenated sesquiterpenes/alcohols with antimicrobial and fragrance properties^59,60^.

The lipid profile of BSO with the simple unsaturated fatty acids as linoleic acid (C18:2), oleic acid (C18:1), and linolenic acid (C18:3) is known for its fluidizing effect on cell membranes, cardioprotective, skin conditioning, and anti-inflammatory^53–55^. While Arachidonic acid, cis-11-Eicosenoic acid, cis-11,14-Eicosadienoic acid, Erucic acid, and *cis*-13,16-Docosadienoic acid are considered long-chain polyunsaturated fatty acids with roles in signaling, metabolism, and skin health^61–64^. Inaddition, the saturated fatty acids of Myristic (C14:0), Palmitic (C16:0), Stearic (C18:0), Behenic (C22:0), and Lignoceric (C24:0) are common in plant oils and influence melting point, skin occlusiveness, shelf-life, and provide oxidative stability and structural integrity^65,66^.

### Nanoemulsion loaded NCh characterization

#### Fourier transform infrared spectroscopy (FTIR)

**Fig. 1:**
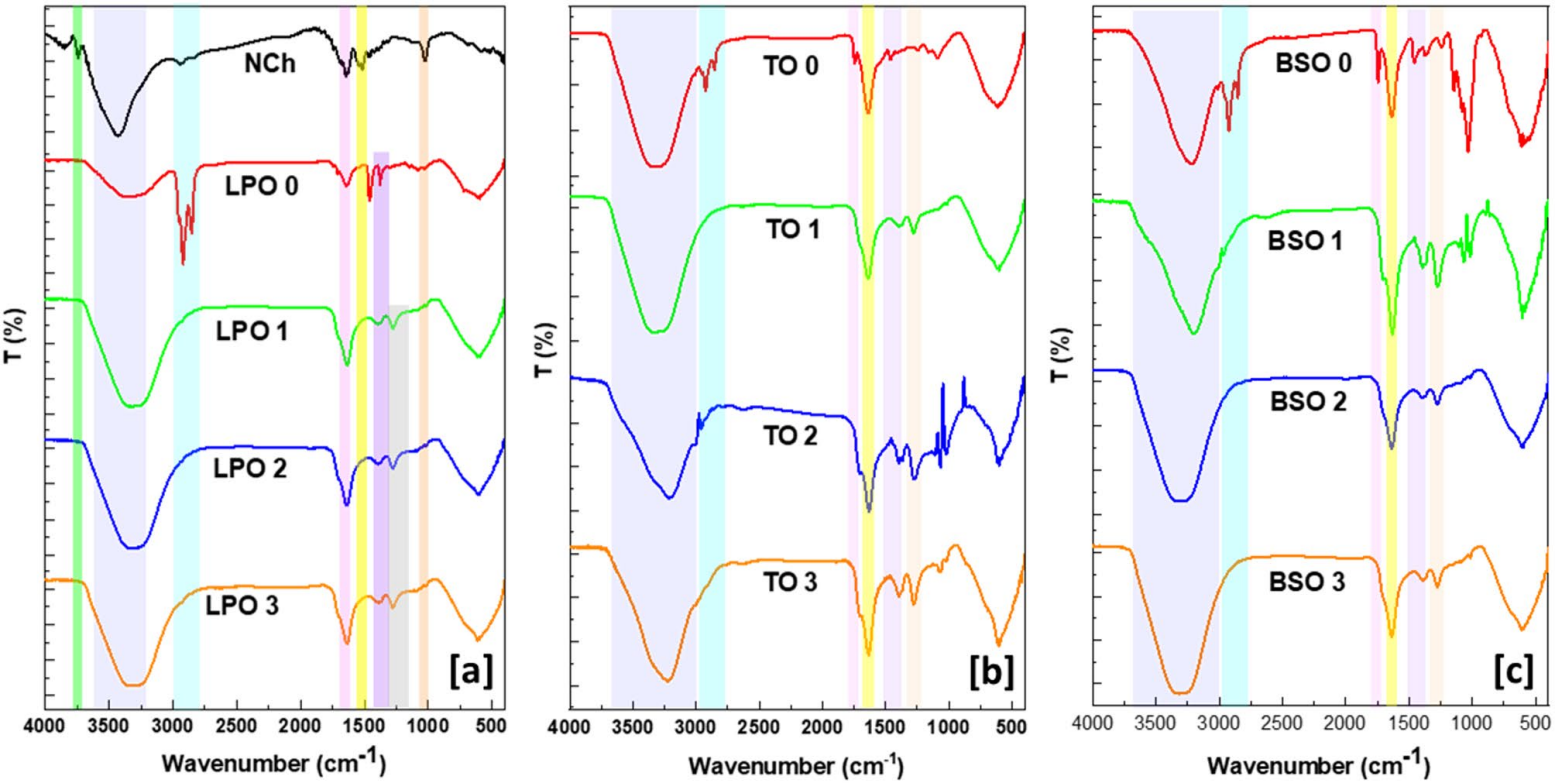
FTIR of NCh (**a**), nanoemulosions loaded nanochitosan. (**a**) PLO, (**b**) TO, and (**c**) BSO. 0, 1, 2, and 3 are represented by the oil concentration as 0, 1, 2, and 3% (*w/v*), respectively.

FTIR spectra of the NCh and nanoemulsions and nanoemulsion-loaded NCh were observed in Fig. 1. NCh (Fig. 1a) assigned characteristic bands at 3746, 3435, 2919, 1635, 1517, and 1022 cm^-1^, corresponding to –NH_2_ groups stretching vibration, –OH groups stretching vibration, CH stretching vibration, CONH_2_ and NH_2_ groups, C-O-C band, respectively^37,67^. In addition, the nanoemulsion was studied individually for each of the EOs. The nanoemulsion of LPO (Fig. 1a) observed characteristic bands at 3232, 2910, 1746, 1639, and 1237 cm^-1^ related to O-H groups stretching vibration, C-H groups stretching vibration, C = O stretching, C = C stretching, and C-O stretching, respectively^68^. Meanwhile, the addition of nanoemulsion to NCh affected the molecular structure of both NCh and EO, as observed in the FTIR spectra^69^. Hence, the –NH_2_ groups and CONH_2_ disappeared, and the OH groups band was shifted to 3185 cm^-1^^70^. Moreover, the CH and C = O bands were reduced to a small band at 2952 and 1711 cm^-1^, respectively^71^. However, the bands C = C and C-O were 1617, 1263 cm^-1^^72^. These observations could be due to the interaction of EO molecules and NH_2_ groups according to their high reactivity^73^. Additionally, incorporating EO into the NCh structure is relevant to this shift and change in the group’s intensity effect. TO samples (Fig. 1b) observed a diaper of the N-H and C-H groups stretching vibration with increased EO concentration.

In contrast, the band of C = O (at around 1717 cm^-1^) appeared with a high concentration of EO. The sample BSO spectra (Fig. 1c) show the typical behavior with a unique broadness in the OH stretching vibration band. Indeed, these observations of each EO affirmed that the NCh FTIR spectrum characteristics bands disappeared completely in a high concentration of nanoemulsion addition due to the dominant effect of oil particles on the molecular structure of the loaded system. Moreover, some differences were observed in the FTIR spectra of each oil, which could be due to the nature and chemical composition of each oil.

#### Dynamic light scattering (DLS)

**Fig. 2:**
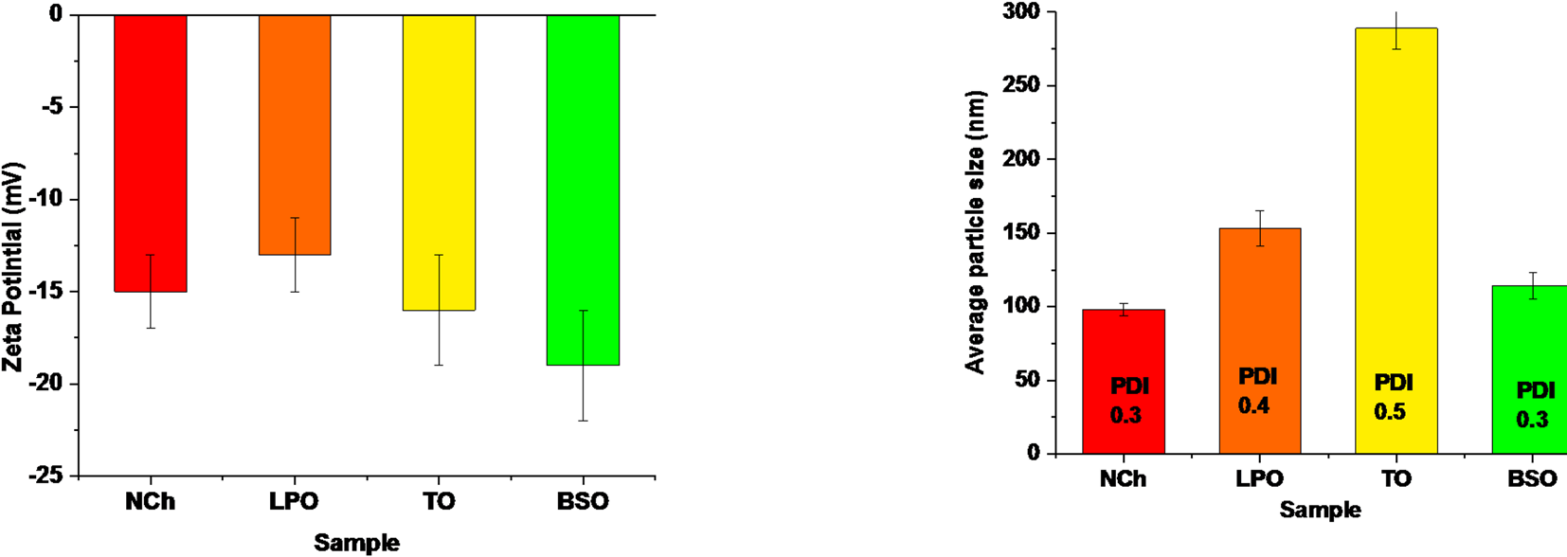
Average zeta potential (left), polydispersity index (PDI), and the average particle size (right) of the formulated NCh and nanoemulsions.

Another part of the physicochemical study is DLS measurements that included particle size distribution, polydispersity index (PDI), and average zeta potential, as illustrated in Fig. 2. These measurements are relevant to particle size, homogeneity, and stability. The average zeta potential of NCh was recorded as − 15 (± 2) mV, which indicates a moderately stable colloidal solution^74^. In addition, the LPO, TO, and BSO nanoemulsion-loaded chitosan samples recorded (individually) − 13 (± 2), − 16 (± 3), and − 19 (± 3) mV, respectively, with good stability for BSO nanoemulsion-loaded chitosan. Conversely, the particle average size of NCh was recorded as 98 (±) nm, which increased after loading with EOs to 153 (± 12), 289 (± 14), and 114 (± 9) nm for LPO, TO, and BSO formulas, respectively. Besides, these were reflected in the PDI value that recorded the highest index for TO as 0.5, which referred to moderate homogeneity, and this is expected according to the particle size and zeta measurement. In addition, the NCh index was recorded as 0.3, which referred to good particle homogeneity, and the LPO and BSO indexes were 0.4 and 0.3, respectively^75^. The difference between the different EOs was due to each oil’s natural and chemical composition^76,77^, and this is the same conclusion of the FTIR study.

#### Transmission electron microscopy (TEM)

**Fig. 3:**
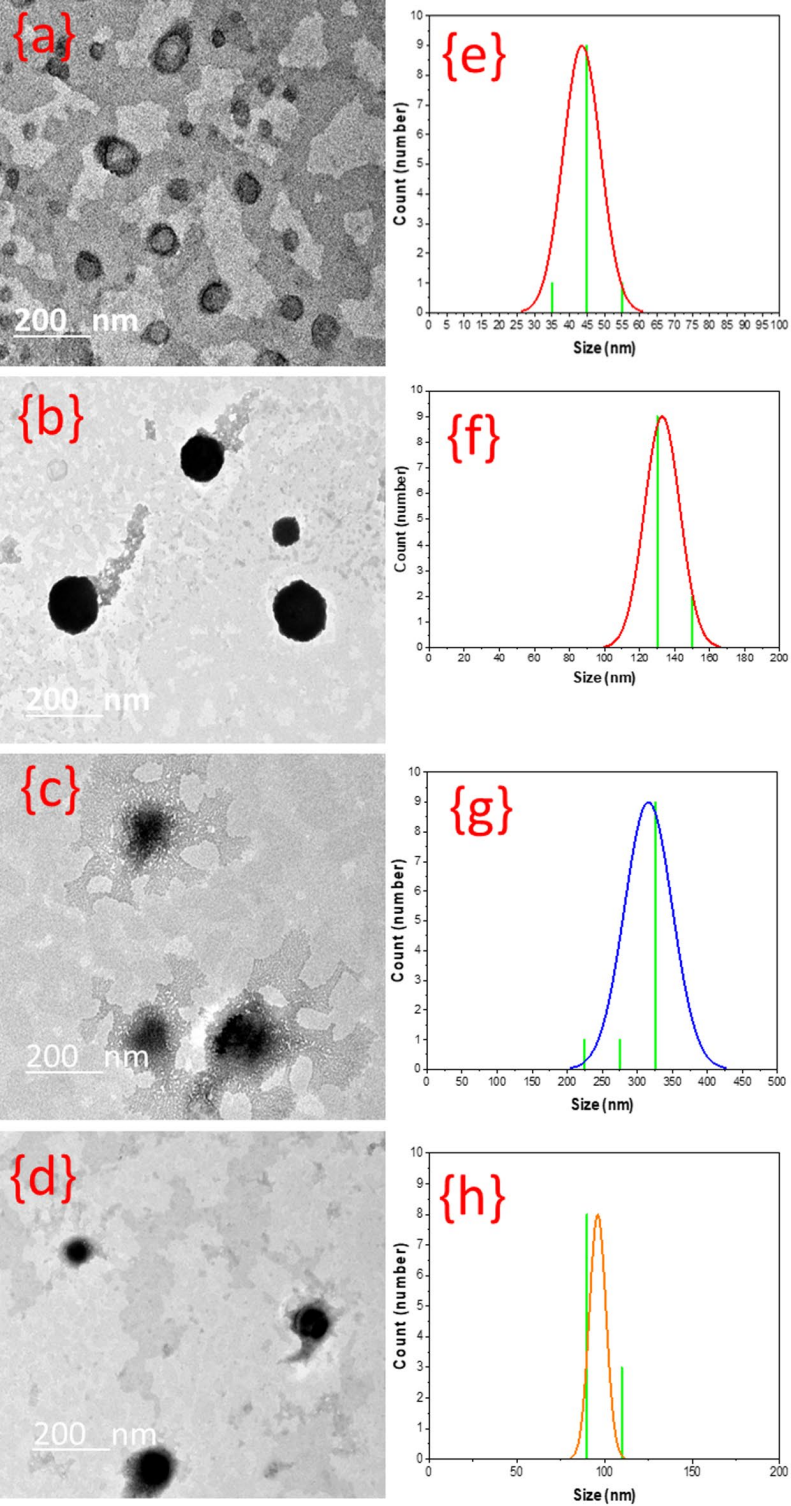
TEM images of NCh (**a**) and nanoemulsions loaded NCh for LPO 2% (**b**), TO 2% (**c**), and BSO 2% (**d**), as well as the calculated particle size distribution histograms for NCh (**e**), LPO 2% (**f**), TO 2% (**g**), and BSO 2% (**h**).

The morphology of the particles was studied using HR-TEM, as illustrated in Fig. 3. Nanochitosan (Fig. 3a) is described as a sphere with particles around 43 nm in a homogenous shape. The particle size distribution histogram of NCh (Fig. 3e) shows that the particle size ranged from 35 to 55 nm, with the main peak at about 45 nm. On the other hand, the nanoemulsions loaded with NCh TEM images were described as typical nanoemulsions with a core and shell that look like a wall with a faint black color and a core with a dark black^78^. In detail, the LPO nanoemulsion loaded NCh (Fig. 3b) appeared as a spherical core with a deep black color and faint shell due to the nanoemulsion components with an average size of around 121 nm. The particle size distribution histogram of LPO (Fig. 3f) presented particles with a size of about 129 nm as the main and some with a size of about 149 nm. The TO nanoemulsion in Fig. 3c was observed with an irregular spherical core, and the shell thickness was increased compared to the above sample. The average size was detected as around 198 with negligible shell thickness and 327 nm with shell. The TO particle size distribution histogram (Fig. 3g) presented the particle size at about 225 nm and 275 nm as low and high counts at 324 nm. The BSO sample in Fig. 2d detected the lowest nanosize for the core and shell, around 97 nm. BSO’s particle size distribution histogram (Fig. 3h) presented the particle size at about 90 nm and some at 110 nm. Indeed, the HR-TEM images emphasized the DLS measurements based on the particle size and PDI. Moreover, the particles of the samples exhibited the same appearance with different sizes, which could result from each EO property supported by the physicochemical and HR-TEM analysis.

### In vitro results

#### Cytotoxicity of essential nanoemulsion loaded Nanochitosan

The cytotoxicity of new compounds is the first step in checking their biosafety toward normal cell lines. In the current study, prepared compounds LPO 3%, TO 3%, BSO 3%, and Nch were assessed for cytotoxicity toward the Wi 38 normal cell line, as illustrated in Table 10. Results revealed that IC_50_ of LPO 3%, TO 3%, BSO 3%, and Nch were 437, 512, 389, and > 1000 µg/ml, respectively. Generally, the material is classified as non-cytotoxic if the IC50 is ≥ 90 µg/mL^79^. Thus, all these compounds are considered safe to use.

**Table 10:** Cytotoxicity of essential nanoemulsion loaded nanochitosan.

Conc. µg/ml	Cell viability %			
	LPO 3%	TO 3%	BSO 3%	Nch
1000	29.80 ± 1.22^d^	34.47 ± 0.81^d^	15.33 ± 0.58^d^	70.47 ± 1.50^c^
500	42.10 ± 0.85^c^	53.67 ± 0.58^c^	35.03 ± 1.00^c^	91.03 ± 0.95^b^
250	73.47 ± 1.47^b^	81.13 ± 1.03^b^	59.33 ± 1.53^b^	98.67 ± 0.58^a^
125	97.47 ± 0.50^a^	98.67 ± 0.58^a^	83.63 ± 1.10^a^	99.17 ± 0.29^a^
IC_50_ µg/ml	437	512	389	> 1000

Letters a, b, c & d mean significance power according to Tukey method.

#### Antifungal activity

##### Antifungal susceptibility of mucorales toward traditional antifungal drugs

Mucorales fungi, the causative agents of mucormycosis, are generally resistant to many azole antifungal agents due to the absence or limited expression of the target enzyme, lanosterol 14α-demethylase. Clinical studies have shown that azole antifungal agents, when used as monotherapy, have limited efficacy in treating invasive mucormycosis^80^. Azole antifungals can cause adverse effects, such as hepatotoxicity, and have significant drug-drug interactions, which can limit their use in patients with multiple comorbidities or concomitant medications.

**Fig. 4:**
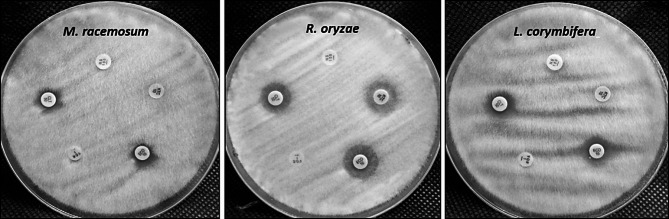
Evaluation of the antifungal activity of commercial antifungal drugs on agar plates.

Clotrimazole, Nystatin, Econazole, Voriconazole, and Ketoconazole antifungal drugs were tested for evaluating the antifungal susceptibility toward M. racemosum, R. microsporus, and *L. corymbifera* as shown in Fig. 4; Table 11. Results revealed that all tested fungal strains were resistant to all tested antifungal discs. This result confirmed that these fungal strains are multidrug-resistant fungi. Thus, alternative antifungal agents are required to tackle these fungi.

**Table 11:** Antifungal susceptibility of *M. racemosum*,* R. microsporus*, and *L. corymbifera* toward commercial antifungal drugs.

Fungal strain	Inhibition zone diameter (mm)				
	Clotrimazole	Nystatin	Econazole	Ketoconazole	Voriconazole
*M. racemosum*	11 (R)	0 (R)	10 (R)	0 (R)	0 (R)
*R. microspores*	13 (R)	0 (R)	14 (R)	13 (R)	0 (R)
*L. corymbifera*	11 (R)	0 (R)	10 (R)	0 (R)	0 (R)

##### Antifungal activity of essential nanoemulsion loaded nanochitosan

The antifungal activity of essential oil nanoemulsions has shown promise in combating invasive fungal infections, a major public health concern due to the increasing incidence and severity, and the limited number of effective antifungal therapies. Several studies have reported the potent antifungal effects of essential oil nanoemulsions against pathogenic fungi, such as Candida species, *Aspergillus* species, and *Cryptococcus neoformans*, which are common causative agents of invasive fungal infections^81,82^. Ongoing research in this area aims to optimize the nanoemulsion formulations further and explore their potential clinical applications in managing invasive fungal infections, which could provide a much-needed alternative to conventional antifungal therapies.

**Fig. 5:**
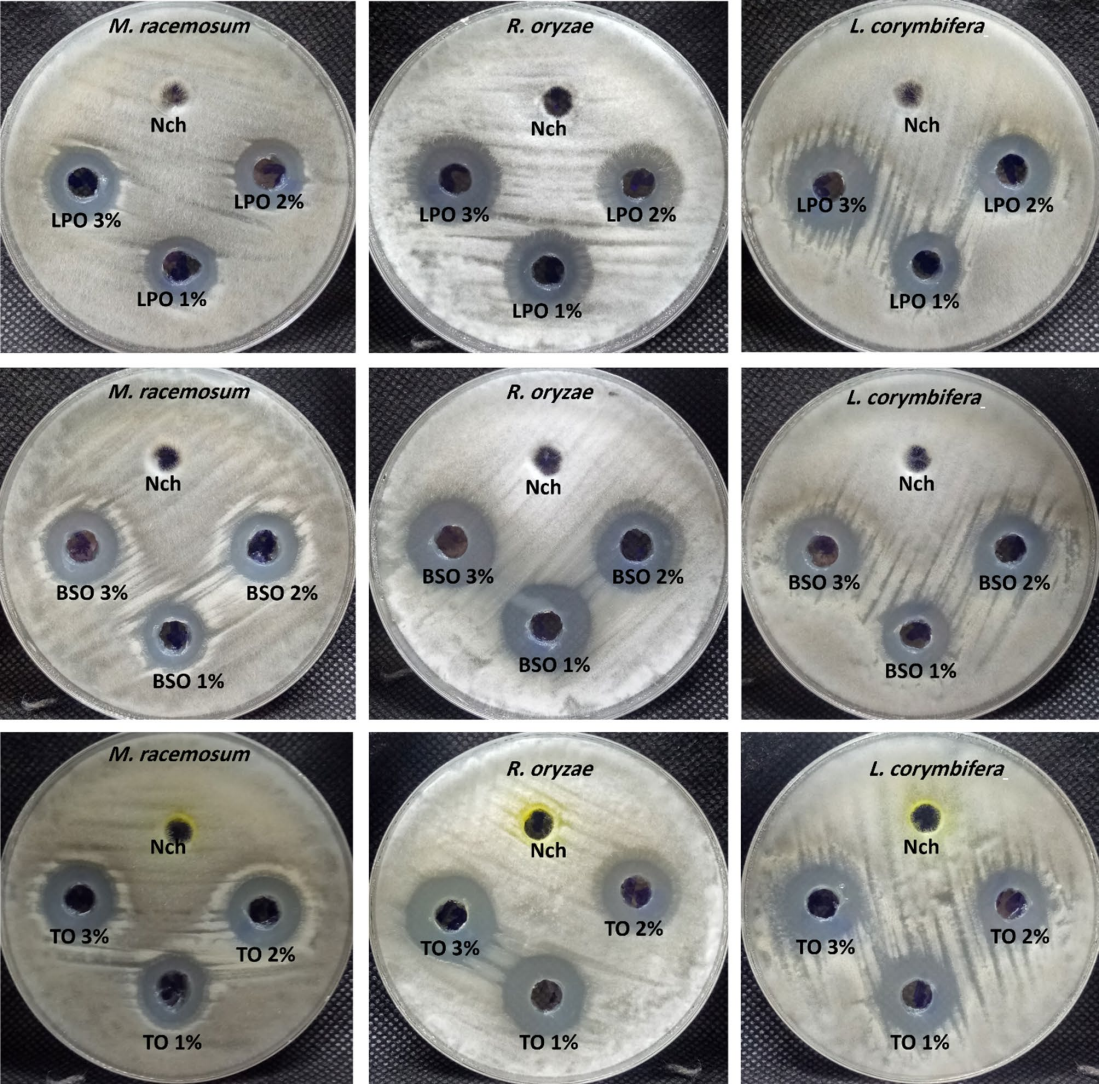
Antifungal activity of nanochitosan loaded LPO 1, 2 & 3%, TO 1, 2 & 3%, BSO 1, 2 & 3%, and NCh only against *M. racemosum*,* R. microsporus*, and *L. corymbifera* fungi using agar well diffusion method.

In the current study, lemon peel, Turmeric essential, and black seed oils were loaded on nanochitosan to enhance the antifungal activity toward some mucorales fungi, which may cause mucormycosis. Essential nanoemulsions loaded with nanochitosan with different concentrations of 1, 2, and 3% were tested for antifungal activity using the agar well diffusion method (Fig. 5). Results revealed that all prepared Essential nanoemulsions loaded with nanochitosan with different concentrations exhibited promising antifungal activity toward *M. racemosum*,* R. microsporus*, and *L. corymbifera* (Table 12). Moreover, lemon peel nanoemulsion-loaded nano chitosan (LPO) showed antifungal activity at 1, 2 & 3% concentrations toward *M. racemosum*,* R. microspores*, and *L. corymbifera*. Furthermore, results illustrated non-significant variations with all concentrations used toward *M. racemosum*,* R. microsporus*, and *L. corymbifera*, where inhibition zones in all treatments range from 17 to 23 mm.

**Table 12:** Antifungal activity of NCh and the prepared NCh-loaded essential oils.

Treatment code	Inhibition zone/mm		
	M. racemosum	*R*. microsporus	L. corymbifera
NCh	ND	ND	ND
LPO (1%)	19.23 ± 0.87^a^	20.9 ± 0.85^b^	18.0 ± 1.0^b^
LPO (2%)	19.4 ± 1.63^a^	21.16 ± 0.76^ab^	19.2 ± 1.16^b^
LPO (3%)	20.0 ± 1.0^a^	22.96 ± 0.85^a^	22.0 ± 1.10^a^
TO (1%)	18.13 ± 1.206^a^	20.0 ± 1.1^b^	20.6 ± 1.04^a^
TO (2%)	19.26 ± 1.419^a^	20.13 ± 1.2^b^	20.13 ± 1.2^a^
TO (3%)	20.0 ± 1.00^a^	23.16 ± 1.25^a^	21.06 ± 1.1^a^
BSO (1%)	17.03 ± 1.35^a^	21.90 ± 1.153^a^	17.20 ± 1.1^a^
BSO (2%)	18.03 ± 1.05^a^	22.0 ± 1.00^a^	18.26 ± 1.4^a^
BSO (3%)	20.06 ± 1.60^a^	23.83 ± 1.04^a^	19.167 ± 1.0^a^

Letters a, b, c, … etc. mean the power significance according to Tukey method.

Essential oils (EOs) are natural, volatile, and complex substances with a highly stimulating odor that aromatic plants produce as secondary metabolites collected and concentrated from various plant areas^83–85^. Also, it showed different phytochemical structures and biological applications^86–88^.

Lemon essential oil and extract have been shown to inhibit the growth of *Aspergillus* species, including *A. fumigatus*, *A. flavus*, and *A. niger*, due to their high content of terpenes and phenolic compounds like limonene, linalool, and citral^89,90^. Lemon extracts have also exhibited antifungal activity against *Candida albicans*, the primary causative agent of candidiasis, through mechanisms involving disruption of cell membrane integrity and suppression of fungal enzymes^91^.

In our findings, monoterpenoid structures were lemon peel’s most predominant EO class. These results may be explained by Hernawan et al., who reported that lemon peel contains terpenoids that can prevent the creation of ergosterol, a component of the fungal cell wall that helps preserve cell membrane permeability. The essential oil from lemon peel is expected to prevent *Candida albicans* growth^92^.

Also, curcumin’s properties have been utilised to explain most of turmeric’s pharmacological effects, as turmeric oil and curcuminoids have yet to be studied. Turmeric rhizome oil (TO) is responsible for flavor and aroma. Dried rhizomes produce approximately 3–6% essential oil. TO has shown in vitro antifungal effectiveness against various fungal strains^93^, as well as a synergistic impact with commercially available azole derivative antifungal medications^93^, such as turmerone and curcumin^94–96^. TO is a viable ecofriendly alternative for reducing fungal contamination in food, as synthetic chemical fungicides negatively affect crops and people^95^. These findings are consistent with the antifungal activity reported in our data and explain that the activity could be attributed to the presence of turmerone structures.

In addition, *Nigella sativa* is a medicinal plant species that has gained attention for a wide range of therapeutic uses^97–100^due to its seeds, which are rich in phytoconstituents such as proteins, carbohydrates, vitamins, dietary minerals (such as Fe and Zn), crude fiber, alkaloids, saponins, steroids, terpenoids, p-cymene, limonene, and fatty acids^101,102^. The phytochemical components and antibacterial activities of natural oil *N. sativa* seeds provide new prospects for finding and developing effective antibiotics as alternative treatments for drug-resistant pathogenic bacterial strains^101,103–106^.

Among the major identified compounds, Linalool, a volatile compound, is documented as an antifungal agent, especially against Candida and Aspergillus species by disrupting the membrane, interfering with ergosterol synthesis, and inducing oxidative stress^107–109^. But, Geraniol monoterpene alcohol had significant antifungal and antibiofilm activity by acting on fungal cell membranes and affecting mitochondrial function^110,111^. At the same time, the oxygenated sesquiterpene of *β*-Tumerone from turmeric exhibits antifungal activity, possibly via ROS generation and cell wall interference^112,113^. Also, Linoleic acid (C18:2), as one of the unsaturated fatty acids, has been reported to have more potent antifungal properties than saturated ones. They disrupted fungal membranes, inhibited growth, and could induce oxidative damage^114,115^. In particular, Linoleic acid was active against *Candida albicans* and dermatophytes^114,116^. On the other hand, the **s**aturated fatty acids and sterols alone are weakly active but may support formulation stability or boost penetration of active compounds^117–120^.

Essential oil nanoemulsions exhibit potent antifungal activity through several fundamental mechanisms: disruption of the fungal cell membrane, leading to increased permeability and leakage of cellular contents; inhibition of critical fungal enzymes involved in growth, metabolism, and virulence, such as ergosterol biosynthesis; induction of oxidative stress within fungal cells, causing damage to lipids, proteins, and DNA; and modulation of fungal signal transduction pathways, altering gene expression and cellular processes important for growth and pathogenicity^121,122^. The small size and high surface area-to-volume ratio of nanoemulsions enhance their ability to interact with and penetrate fungal cells. This enables these multifaceted antifungal effects, further amplifying the complex mixture of essential oil compounds’ synergistic interactions^123^.

## Conclusion

In this study, three herbal oils were developed through different formulas using the prepared NCh and were evaluated for their antifungal properties against fungi causing mucormycosis. Formulated nanoemulsions-based EOs were successfully prepared and affirmed using physicochemical and HR-TEM analyses. Phytochemical analysis identified the key chemical constituents of the essential oils, including oxygenated/non-oxygenated hydrocarbons and saturated/unsaturated fatty acids. Physicochemical characterization confirmed the formation of stable nanoemulsions with desirable particle size, distribution, and surface charge properties. The nanostructure was assigned for LPO, TO, and BSO. Moreover, EOs affect the size of particles in core-shell dimensions. Notably, the essential oil nanoemulsions exhibited potent antifungal activity against the tested fungal strains, outperforming commercial antifungal drugs to which these fungi were found to be resistant. The 1–3% chitosan-based nanoemulsions demonstrated inhibition zones ranging from 17 to 23 mm, indicating their promising potential as natural and eco-friendly antifungal agents. Limitations of the current study include insufficient in vitro conditions; thus, in vivo studies are required. Additionally, the mechanisms of antifungal action were not explored, and comprehensive safety evaluations in vivo are necessary. Finally, the economic feasibility of large-scale production remains uncertain, potentially limiting practical applications.

In this study, three herbal oil formulations were developed using prepared nanochitosan (NCh) and evaluated for their antifungal efficacy against fungi responsible for mucormycosis. Nanoemulsion-based essential oils (EOs) were successfully formulated and validated through physicochemical characterization and high-resolution transmission electron microscopy (HR-TEM). Phytochemical analysis revealed the major constituents of the essential oils, including oxygenated and non-oxygenated hydrocarbons and saturated and unsaturated fatty acids. The physicochemical data confirmed the formation of stable nanoemulsions with favorable particle size, distribution, and surface charge. Nanostructures were identified for lemon peel oil (LPO), turmeric oil (TO), and black seed oil (BSO), with essential oils influencing the particle morphology within core-shell dimensions. Importantly, the EO-based nanoemulsions exhibited strong antifungal activity against resistant fungal strains, surpassing the efficacy of conventional antifungal drugs. Chitosan-based nanoemulsions at 1–3% produced inhibition zones ranging from 17 to 23 mm, highlighting their potential as natural and environmentally friendly antifungal agents. However, the study is limited by its in vitro design, necessitating further in vivo investigations. Additionally, the specific mechanisms of antifungal action were not examined, and comprehensive safety assessments remain pending. Lastly, the cost-effectiveness of large-scale production has yet to be determined, which may pose challenges for practical application.

## Data Availability

The datasets analyzed during the current study are available in the NCBI GenBank database repository with the accession number of MG547571.1, MK623262.1 and MK300698.1. https://www.ncbi.nlm.nih.gov/nuccore/MG547571; https://www.ncbi.nlm.nih.gov/nuccore/MK623262; https://www.ncbi.nlm.nih.gov/nuccore/MK300698.
